# Transcriptional Profiling Confirms the Therapeutic Effects of Mast Cell Stabilization in a Dengue Disease Model

**DOI:** 10.1128/JVI.00617-17

**Published:** 2017-08-24

**Authors:** Juliet Morrison, Abhay P. S. Rathore, Chinmay K. Mantri, Siti A. B. Aman, Andrew Nishida, Ashley L. St. John

**Affiliations:** a Center for Infection and Immunity, Mailman School of Public Health, Columbia University, New York, New York, USA; b Program in Emerging Infectious Diseases, Duke-National University of Singapore, Singapore; c Department of Pathology, Duke University Medical Center, Durham, North Carolina, USA; d Department of Microbiology, University of Washington, Seattle, Washington, USA; e Department of Microbiology and Immunology, Young Loo Lin School of Medicine, National University of Singapore, Singapore; University of Southern California

**Keywords:** dengue fever, dengue virus, interferons, mast cell, transcriptional regulation

## Abstract

There are no approved therapeutics for the treatment of dengue disease despite the global prevalence of dengue virus (DENV) and its mosquito vectors. DENV infections can lead to vascular complications, hemorrhage, and shock due to the ability of DENV to infect a variety of immune and nonimmune cell populations. Increasingly, studies have implicated the host response as a major contributor to severe disease. Inflammatory products of various cell types, including responding T cells, mast cells (MCs), and infected monocytes, can contribute to immune pathology. In this study, we show that the host response to DENV infection in immunocompetent mice recapitulates transcriptional changes that have been described in human studies. We found that DENV infection strongly induced metabolic dysregulation, complement signaling, and inflammation. DENV also affected the immune cell content of the spleen and liver, enhancing NK, NKT, and CD8^+^ T cell activation. The MC-stabilizing drug ketotifen reversed many of these responses without suppressing memory T cell formation and induced additional changes in the transcriptome and immune cell composition of the spleen, consistent with reduced inflammation. This study provides a global transcriptional map of immune activation in DENV target organs of an immunocompetent host and supports the further development of targeted immunomodulatory strategies to treat DENV disease.

**IMPORTANCE** Dengue virus (DENV), which causes febrile illness, is transmitted by mosquito vectors throughout tropical and subtropical regions of the world. Symptoms of DENV infection involve damage to blood vessels and, in rare cases, hemorrhage and shock. Currently, there are no targeted therapies to treat DENV infection, but it is thought that drugs that target the host immune response may be effective in limiting symptoms that result from excessive inflammation. In this study, we measured the host transcriptional response to infection in multiple DENV target organs using a mouse model of disease. We found that DENV infection induced metabolic dysregulation and inflammatory responses and affected the immune cell content of the spleen and liver. The use of the mast cell stabilization drug ketotifen reversed many of these responses and induced additional changes in the transcriptome and immune cell repertoire that contribute to decreased dengue disease.

## INTRODUCTION

Dengue virus (DENV) is the most common cause of mosquito-borne viral infections in the world. Infection with DENV can cause a spectrum of symptoms in humans, ranging from asymptomatic disease to severe vascular complications known as dengue hemorrhagic fever (DHF) and dengue shock syndrome (DSS) (1). Recent World Health Organization (WHO) guidelines categorize dengue disease into three types: dengue, dengue plus warning signs, and severe dengue (1). There are no specific therapeutics approved for DHF/DSS, and palliative care is currently the only treatment option. DHF/DSS usually occurs at the defervescence phase of disease as viremia subsides and can have severe or fatal outcomes due to vascular hemorrhaging and plasma loss (2). Since virus levels are very low or undetectable at this stage of disease, host factors are thought to play a crucial role in the development of severe dengue. DENV replicates in nonimmune cells such as endothelial cells and fibroblasts as well as in immune cells, including dendritic cells (DCs) and macrophages (3–7). As such, dengue disease involves multiple tissues and organs, such as liver and spleen (8, 9). The capillary leakage, thrombocytopenia, and liver damage that occur with severe disease are accompanied by changes in the blood transcriptome, proteome, and metabolome (10–18). DENV infection is associated with metabolic dysregulation and with increases in levels of inflammatory mediators such as CCL2 (C-C motif chemokine ligand 2), CXCL9 (C-X-C motif chemokine ligand 9), CXCL10, and VEGFA (vascular endothelial growth factor A) (10, 19–21).

Historically, it was thought that productive DENV infections were restricted to humans and other primates and could not occur in immunocompetent mice. This led to the development of several immunodeficient mouse models of infection, which have improved our understanding of some aspects of dengue disease but have not allowed us to investigate the contribution of the host response in the context of an intact innate immune response (22, 23). We recently described an immunocompetent model of dengue disease in which C57BL/6 mice infected intraperitoneally (i.p.) with a DENV clinical isolate, EDEN2, showed symptoms such as thrombocytopenia, increased hematocrit values, vascular leakage, and viral replication in the spleen and liver, which are reminiscent of human disease (24, 25).

Mast cells (MCs) have been identified as inducers of vascular leakage during DENV infection (24, 25). MCs are innate immune cells that line blood vessels and release vasoactive mediators such as tumor necrosis factor alpha (TNF-α) as well as the MC-specific proteases tryptase and chymase upon activation by DENV. Moreover, treatments of animals using MC stabilizers such as ketotifen and cromolyn are effective at limiting DENV-induced vascular leakage as measured by Evans blue and hematocrit assays (24). Mechanistically, ketotifen and cromolyn work by preventing the release of MC granules as well as other soluble mediators such as leukotrienes and platelet-activating factor (26–31). A human clinical trial is under way to evaluate the effectiveness of ketotifen in inhibiting vascular leakage during DENV disease. Ketotifen has a favorable safety profile and is clinically approved for the treatment of asthma and allergic conjunctivitis via the oral and ophthalmic routes, respectively (32). Although ketotifen directly inhibits the release of MC products by inhibiting degranulation, the global impact of MC stabilization during DENV disease was unknown until now.

In this study, we use gene expression profiling of the liver and spleen in an immunocompetent mouse model to address the global molecular impact of MC stabilization during DENV disease. We show that the host response to DENV infection in immunocompetent mice recapitulates many of the transcriptional changes that have been described in human studies. DENV infection increased inflammatory responses and metabolic dysregulation and affected the immune cell content of the spleen and liver. MC stabilization reversed many of these responses and induced additional changes in the transcriptome and cellular content of the liver and spleen.

## RESULTS

### The host response to DENV2 infection is dominated by metabolic dysregulation and inflammatory cytokine signaling.

**FIG 1:**
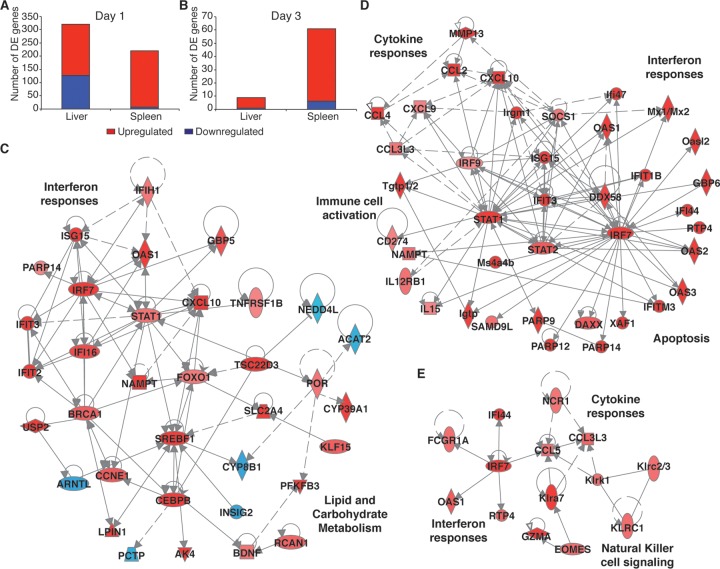
Metabolic dysregulation and inflammatory cytokine signaling dominate the host response to DENV2 infection. (A and B) Numbers of DE genes in DENV-infected livers and spleens on day 1 (A) and day 3 (B). (C) Day 1 network of DE interferon response and metabolism genes in the liver. (D and E) Network of immune cell activation, interferon response, and cytokine response genes in the spleen on day 1 (D) and day 3 (E) post-DENV infection. Criteria used for differential expression analysis were an adjusted *P* value of <0.05, as determined by the limma empirical Bayes-moderated *t* test, and a log_2_ FC of >0.58. Blue represents downregulation, red represents upregulation, and gray represents no differential expression.

To identify the host responses that were induced by DENV2 infection, we compared liver and spleen gene expression levels on days 1 and 3 postinfection in DENV2-infected mice versus mock-infected animals. These time points were chosen since they coincide with the peak time points of virus replication and DENV symptoms such as vascular leakage and thrombocytopenia in this mouse model (24). Active DENV2 replication in target organs was confirmed in this study by the quantitation of total DENV genome copy numbers (see Fig. S1A and S1B in the supplemental material) and negative-strand viral RNA PCR (Fig. S1C). We found that DENV2 infection resulted in the differential expression of 320 genes in the liver on day 1 but that the response in this organ was mostly resolved by day 3, when only 9 differentially expressed (DE) genes were detected (Fig. 1A and B). The liver response was enriched for various metabolic pathways and interferon (IFN) signaling on day 1 (Table 1). Carbohydrate metabolism genes such as SLC2A4 (solute carrier family 2 member 4), PFKFB3 (6-phosphofructo-2-kinase/fructose-2,6-biphosphatase 3), and RCAN1 (regulator of calcineurin 1) were upregulated, as were lipid metabolism genes such as NAMPT (nicotinamide phosphoribosyltransferase), SREBF1 (sterol regulatory element binding transcription factor 1), and LPIN1 (lipin 1) (Fig. 1C). DENV2 infection also induced the upregulation of interferon-stimulated genes (ISGs) such as IFIT3 (IFN-induced protein with tetratricopeptide repeats 3), ISG15, and OAS (2′-5′-oligoadenylate synthetase 1) (Fig. 1C). These ISGs have been shown to promote antiviral activity against DENV using *in vitro* model systems (33–35). On day 3, only two pathways, granzyme A signaling and protein kinase A signaling, were enriched (Table 1), suggesting a transition from an innate immune response toward a robust T cell response for clearing virus infection. The small number of upregulated genes, including GZMA (granzyme A), SPIC (Spi-C transcription factor), and Ly6a (lymphocyte antigen 6 complex, locus A), were associated with immune functions.

**TABLE 1:** Top host pathways that are perturbed after DENV2 infection^*a*^

Organ	Day	Top host pathway	*P* value
Liver	1	Spermidine biosynthesis I	1.51E−04
		Circadian rhythm signaling	6.95E−04
		Interferon signaling	9.72E−04
		Activation of IRF by cytosolic pattern recognition receptors	9.96E−04
		Salvage pathways of pyrimidine ribonucleotides	1.03E−03
		Adipogenesis pathway	1.38E−03
		Folate polyglutamylation	1.48E−03
		Salvage pathways of pyrimidine deoxyribonucleotides	4.03E−03
		Unfolded protein response	4.39E−03
		AMPK signaling	9.13E−03
	3	Granzyme A signaling	7.59E−03
		Protein kinase A signaling	1.39E−01
Spleen	1	Activation of IRF by cytosolic pattern recognition receptors	7.79E−13
		Interferon signaling	3.59E−12
		Role of pattern recognition receptors in recognition of bacteria and viruses	3.63E−10
		Granulocyte adhesion and diapedesis	7.05E−07
		Role of RIG1-like receptors in antiviral innate immunity	1.47E−05
		Death receptor signaling	5.73E−05
		Agranulocyte adhesion and diapedesis	7.49E−05
		Retinoic acid-mediated apoptosis signaling	8.19E−05
		UVA-induced MAPK signaling	1.02E−05
		Role of hypercytokinemia/hyperchemokinemia in the pathogenesis of influenza	2.67E−04
	3	Role of pattern recognition receptors in recognition of bacteria and viruses	6.10E−05
		Natural killer cell signaling	9.16E−04
		Communication between innate and adaptive immune cells	8.59E−03
		Rapoport-Luebering glycolytic shunt	9.38E−03
		Pathogenesis of multiple sclerosis	1.40E−02
		Differential regulation of cytokine production in macrophages and T helper cells by IL-17A and IL-17F	2.79E−02
		Granzyme A signaling	3.09E−02
		Granulocyte adhesion and diapedesis	3.14E−02
		Agranulocyte adhesion and diapedesis	3.54E−02
		Differential regulation of cytokine production in intestinal epithelial cells by IL-17A and IL-17F	3.55E−02

a DE genes from Fig. 1 were analyzed by Ingenuity Pathway Analysis (IPA) to identify the top 10 enriched pathways in the spleen and liver that were perturbed by DENV infection. IRF, interferon regulatory factor; AMPK, AMP-activated protein kinase; MAPK, mitogen-activated protein kinase.

The kinetics of the host response in the spleen were similar to those in the liver, with the greatest number of gene expression changes occurring on day 1. Two hundred twenty DE genes were detected in the spleen on day 1, while 61 DE genes were detected on day 3 (Fig. 1A and B). The top most enriched pathways in the spleen were associated with pathogen recognition, IFN and cytokine responses, and immune cell activation on day 1 (Table 1). Cytokine genes such as CCL2, CCL4, IL-15 (interleukin 15), and CXCL10 were upregulated in the spleen (Fig. 1D). These pathways, along with immune cell signaling pathways, were the most perturbed in the spleen on day 3 (Table 1). For example, natural killer cell signaling genes such as KLRA6 (killer cell lectin-like receptor subfamily A6), KLRC1 (killer cell lectin-like receptor C1), and NCR1 (natural cytotoxicity-triggering receptor 1) were highly expressed in the spleen on day 3 (Fig. 1E). Overall, these data suggest active virus replication and systemic infection followed by the clearance of DENV in this wild-type (WT) mouse model, which are consistent with the viral kinetics reported previously for DENV2 in WT mice (24). Pathways enriched in both the spleens and livers of DENV-infected mice were consistent with those identified in human *in vivo* and *in vitro* studies (10–12, 14, 36, 37).

### Ketotifen treatment reverses DENV2-induced host responses.

**FIG 2:**
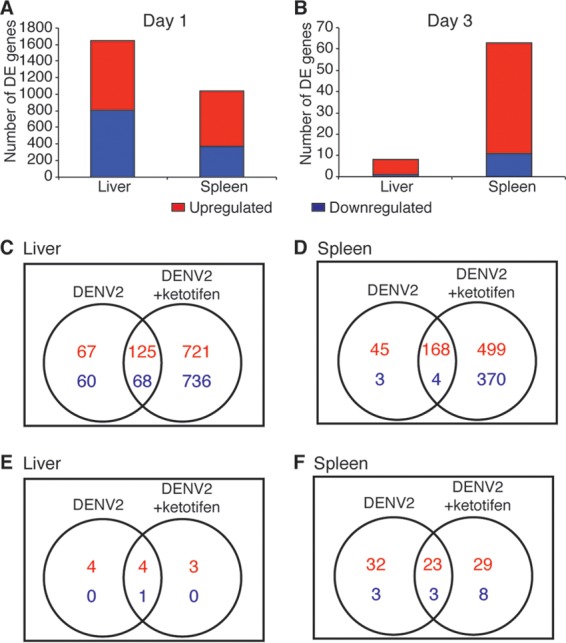
Ketotifen treatment induces a robust host response in DENV2-infected livers and spleens. (A and B) DE genes in DENV-infected and ketotifen-treated organs on day 1 (A) and on day 3 (B). (C to F) Overlaps in the numbers of DE genes between DENV-infected and DENV-infected, ketotifen-treated samples from liver (C and E) and spleen (D and F) on day 1 (C and D) and day 3 (E and F). Criteria used for differential expression analysis were an adjusted *P* value of <0.05, as determined by the limma empirical Bayes-moderated *t* test, and a log_2_ FC of >0.58.

To investigate the effect of MC stabilization on DENV-induced host responses, we transcriptionally profiled the spleens and livers of DENV2-infected mice that had been treated with ketotifen. Animals received intraperitoneal injections of ketotifen or saline daily. The first dose was administered 1 h after infection, with daily drug injections thereafter at 24-h intervals. Although ketotifen treatment did not significantly affect DENV2 replication (see Fig. S1 in the supplemental material), it had a striking effect on the host response to DENV2 infection (Fig. 2). Ketotifen-treated, DENV2-infected mice had a more robust transcriptional response than did untreated, DENV2-infected mice (Fig. 2A and B). Approximately 12% of the liver genes and 18% of the spleen genes that were DE in ketotifen-treated, DENV2-infected mice were also DE in untreated, DENV2-infected mice (Fig. 2C to F).

To probe the responses that were specific to DENV2 infection, we focused on the genes that were DE in untreated, DENV2-infected mice and asked whether their expression was also affected in ketotifen-treated, DENV2-infected mice. We found that ketotifen treatment dampened the DENV-induced gene expression changes by at least 10% for approximately 55% of the genes on day 1 and 67% of the genes on day 3 in the liver (Fig. S2). In the spleen, ketotifen treatment reversed the DENV2-induced expression changes by at least 10% for approximately 45% of the genes on day 1 and 72% of the genes on day 3 (Fig. S3).

### Ketotifen treatment leads to perturbations in cholesterol biosynthesis, intrinsic prothrombin signaling, complement signaling, and LXR/RXR activation in DENV-infected mice.

**FIG 3:**
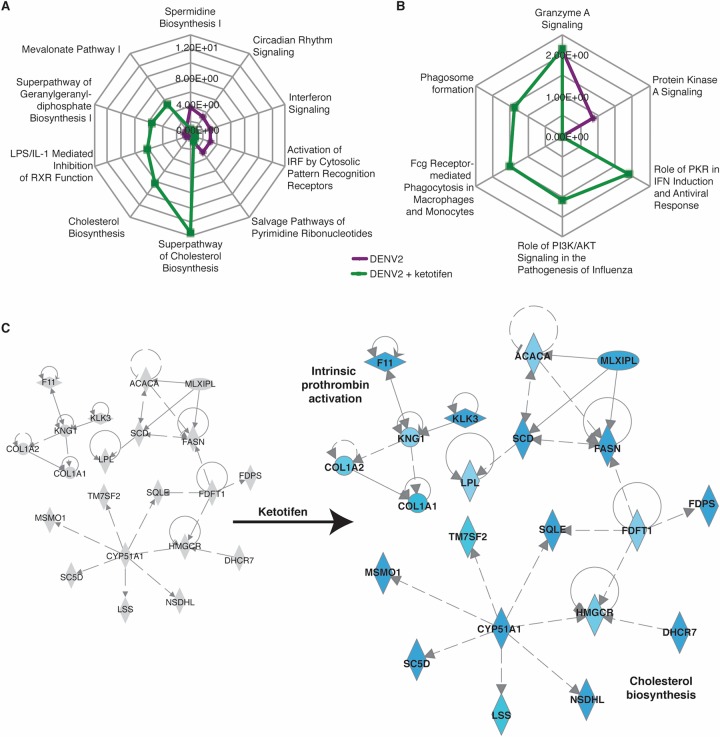
Ketotifen perturbs multiple pathways in the liver. The liver DE genes from Fig. 1 and 2 were analyzed by IPA to produce lists of host pathways that were most perturbed by DENV infection with or without ketotifen treatment. (A and B) The top 10 enriched pathways on day 1 (A) and day 3 (B) are represented as radial plots. The distance from the center in each radial plot represents the enrichment score, which is defined as −log_10_(*P* value), using a right-tailed Fisher exact test. (C) IPA network showing interactions between molecules involved in cholesterol biosynthesis and those involved in intrinsic prothrombin activation on day 1. Blue represents downregulation, red represents upregulation, and gray represents no differential expression.

We next investigated the host pathways that were induced or inhibited by ketotifen. The responses that were most perturbed in the livers of ketotifen-treated, DENV2-infected mice were cholesterol and lipid metabolism pathways on day 1 (Table 2 and Fig. 3). Cholesterol biosynthesis genes such as DHCR24 (24-dehydrocholesterol reductase), CYP51A1 (cytochrome P450 family 51), SQLE (squalene epoxidase), and FDPS (farnesyl diphosphate synthase) were downregulated on day 1 relative to untreated, DENV2-infected animals (Fig. 3C). We also observed decreases in the expression levels of genes such as COL1A1 (collagen type 1 alpha 1), COL1A2, F11 (coagulation factor XI), and KNG1 (kininogen 1) that are involved in intrinsic prothrombin activation (Fig. 3C).

**TABLE 2:** Top host pathways that are perturbed after DENV2 infection and ketotifen treatment^*a*^

Organ	Day	Top host pathway(s)	*P* value
Liver	1	Superpathway of cholesterol biosynthesis	2.55E−14
		Cholesterol biosynthesis I, II, and III	6.90E−09
		LPS/IL-1-mediated inhibition of RXR function	6.87E−07
		Superpathway of geranylgeranyldiphosphate biosynthesis I (via mevalonate)	2.92E−06
		Mevalonate pathway I	4.00E−06
		LXR/RXR activation	1.40E−05
		Estrogen biosynthesis	1.52E−05
		PXR/RXR activation	3.21E−05
		FXR/RXR activation	8.30E−05
		GADD45 signaling	8.48E−05
	3	Granzyme A signaling	6.64E−03
		Role of PKR in interferon induction and antiviral response	1.32E−02
		Role of PI3K/AKT signaling in the pathogenesis of influenza	2.50E−02
		Fcγ receptor-mediated phagocytosis in macrophages and monocytes	3.06E−02
		Phagosome formation	3.99E−02
		Role of NFAT in regulation of the immune response	5.89E−02
		Dendritic cell maturation	6.17E−02
		Systemic lupus erythematosus signaling	7.24E−02
		Glucocorticoid receptor signaling	9.12E−02
		Role of macrophages, fibroblasts, and endothelial cells in rheumatoid arthritis	9.77E−02
Spleen	1	Hepatic fibrosis/hepatic stellate cell activation	4.97E−08
		Complement system	9.90E−08
		LXR/RXR activation	2.22E−07
		Role of pattern recognition receptors in recognition of bacteria and viruses	1.57E−06
		Cholesterol biosynthesis I, II, and III	4.65E−06
		Cell cycle control of chromosomal replication	5.88E−06
		LPS/IL-1-mediated inhibition of RXR function	6.07E−06
		Cross talk between dendritic cells and natural killer cells	8.39E−06
		Death receptor signaling	1.24E−05
		Pyrimidine deoxyribonucleotide *de novo* biosynthesis I	1.88E−05
	3	Interferon signaling	7.10E−05
		Role of pattern recognition receptors in recognition of bacteria and viruses	2.48E−04
		Activation of IRF by cytosolic pattern recognition receptor	3.61E−04
		Communication between innate and adaptive immune cells	1.04E−03
		Role of RIG1-like receptors in antiviral innate immunity	4.17E−03
		Granulocyte adhesion and diapedesis	7.25E−03
		Agranulocyte adhesion and diapedesis	8.67E−03
		Chemokine signaling	1.10E−02
		Pathogenesis of multiple sclerosis	1.99E−02
		Differential regulation of cytokine production in macrophages and T helper cells by IL-17A and IL-17F	3.95E−02

a DE genes from Fig. 1 were analyzed by IPA to identify the top 10 enriched pathways in the spleen and liver that were perturbed by DENV infection and ketotifen treatment. LPS, lipopolysaccharide; PXR, pregnane X receptor; FXR, farnesoid X receptor; PKR, eukaryotic translation initiation factor 2 alpha kinase 2 (EIF2AK2); PI3K, phosphatidylinositol 3-kinase; NFAT, nuclear factor of activated T cells.

**FIG 4:**
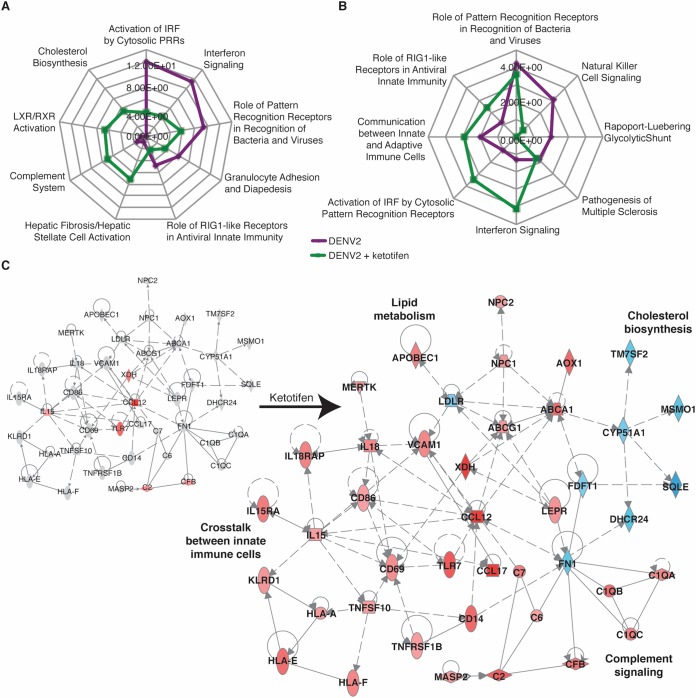
Ketotifen perturbs multiple pathways in the spleen. The spleen DE genes from Fig. 1 and 2 were analyzed by IPA to produce lists of host pathways that were most perturbed by DENV infection with or without ketotifen treatment. (A and B) The top 10 enriched pathways at day 1 (A) and day 3 (B) are represented as radial plots. The distance from the center in each radial plot represents the enrichment score, which is defined as −log_10_(*P* value), using a right-tailed Fisher exact test. PRRs, pattern recognition receptors. (C) IPA network showing interactions between molecules involved in cholesterol biosynthesis, complement signaling, lipid metabolism, and cross talk between immune cells on day 1. Blue represents downregulation, red represents upregulation, and gray represents no differential expression.

Perturbation of cholesterol biosynthesis, complement signaling, and liver X receptor (LXR)/retinoid X receptor (RXR) pathways dominated the splenic responses in ketotifen-treated, DENV2-infected mice on day 1 (Table 2 and Fig. 4). LXR is activated by oxysterol ligands and forms a heterodimer with RXR to initiate the transcription of target genes. LXR/RXR signaling is important for the regulation of lipid metabolism, inflammation, and cholesterol catabolism (38). Cholesterol biosynthesis genes such as DHCR24, CYP51A1, and SQLE were downregulated, but other lipid metabolism genes such as ABCG1 (ATP binding cassette subfamily G member 1) and ABCA1 were upregulated (Fig. 4C). Ketotifen also promoted the expression of complement genes, such as C2, C6, and C1QA, as well as genes that are required for cross talk between innate and adaptive immune cells and the detection of pathogens by lymphocytes, such as CD69, CD86, HLA-A (major histocompatibility complex, class I, A), IL-18, and KLRD1 (Fig. 4C).

### Ketotifen treatment decreases inflammation while preserving the antiviral capacity of immune cells in DENV-infected mice.

**FIG 5:**
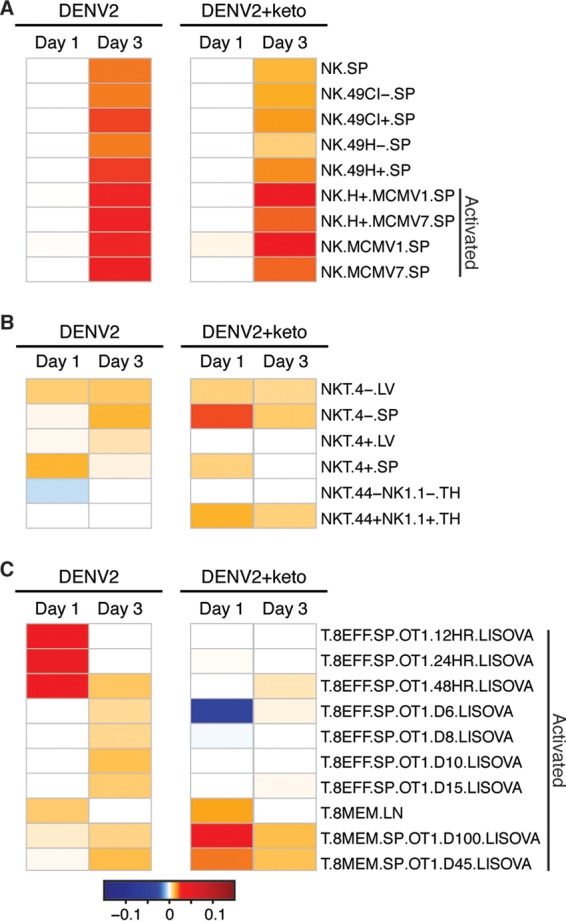
Ketotifen treatment is predicted to modulate lymphocyte numbers in the spleen. The tissue deconvolution-based DCQ algorithm was used to infer immune cell quantities from gene expression changes in the spleen. Enriched natural killer (A), NKT (B), and CD8^+^ (C) cell populations in spleens of mice infected with DENV2 with or without ketotifen treatment are presented as heat maps.

Transcriptional profiling revealed that both DENV infection and ketotifen treatment perturbed innate and adaptive immune cell signaling pathways in the liver and spleen, suggesting that immune cell population changes were occurring in these organs. MCs are known to recruit NKT cells in the skin to help clear peripheral DENV infection (39). However, the recruitment of these cells to systemic sites like the spleen may lead to inflammation and associated pathology. To characterize the effect of ketotifen treatment on immune cell recruitment and inflammation, we employed a tissue deconvolution algorithm that uses whole-organ gene expression data as its input. The digital cell quantification (DCQ) algorithm predicts the quantities of immune populations in an organ by pairing mouse immune cell transcriptional profiles curated by ImmGen (Immunological Genome Project) with whole-organ transcriptional profiles (40). ImmGen is a public resource that contains a compendium of microarray data derived from mouse immune cell populations isolated under standardized conditions. Many of the cell types profiled in ImmGen were isolated from the spleen under steady-state conditions or after perturbations such as bacterial or viral infection. Dengue disease is characterized by acute increases in the numbers of cytotoxic NK cells, NKT cells, and CD8^+^ T cells (41–44). DCQ predicted increases in the numbers of splenic NKT, NK, and CD8^+^ T cells after DENV infection (Fig. 5). DENV infection was predicted to increase the numbers of NK cells of the Ly49H^+/−^ and Ly49C/I^+/−^ subsets, and ketotifen was predicted to reduce this increase (Fig. 5A). These splenic NK cells were predicted to be activated, as they are transcriptionally similar to NK cells isolated after viral infection (Fig. 5A).

Numbers of splenic CD4^+/−^ NKT cells were also predicted to be increased after DENV infection. Although ketotifen treatment was predicted to reduce the DENV-induced increase in the number of CD4^+^ NKT cells, it was projected to increase CD4^−^ NKT cell quantities. CD44^+^ and CD44^−^ NKT cells were also differentially affected by ketotifen (Fig. 5B). Ketotifen treatment was projected to reverse the DENV-mediated reduction in the quantities of CD44^−^ NK1.1^−^ NKT cells while increasing the quantities of CD44^+^ NK1.1^+^ NKT cells (Fig. 5B). The numbers of effector CD8^+^ T cells were predicted to increase after DENV2 infection, and ketotifen treatment was predicted to reduce the numbers of these cells with effector phenotypes (Fig. 5C). Thus, ketotifen was predicted to increase the number of memory cells in both the NKT and CD8^+^ T cell populations in infected animals relative to vehicle treatment of infection.

**FIG 6:**
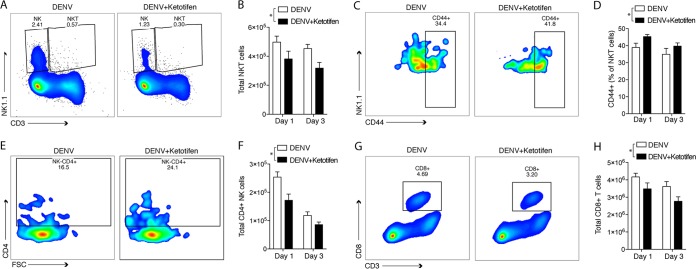
Flow cytometry validates DCQ predictions. Flow cytometry was performed to validate that DCQ correctly interpreted the frequencies of lymphocytes that were differentially enriched or activated during DENV infection and/or ketotifen treatment. (A) Representative plots from day 3 showing the percentages of NK and NKT cells in the spleen. (B) Quantitation of NKT cells from panel A. (C) Representative plots showing the quantities of CD44^+^ NKT cells on day 3. (D) Percent expression of CD44 on NKT cells corresponding to data shown in panel C (*n* = 5). (E) Representative plots showing the percentages of NK cells that are CD4^+^ on day 1. (F) Quantitation of activated NK cells (NK1.1^+^ CD4^+^) on days 1 and 3. (G) Representative plots from day 3 showing the percentages of CD8^+^ T cells in the spleen. (H) Quantitation of CD8^+^ T cells from panel G (*n* = 5). For all panels containing graphs (*n* = 5), analysis was performed by 2-way analysis of variance (* indicates a *P* value of <0.05).

To further assess the influence of ketotifen on splenic inflammation and validate whether the DCQ algorithm reflected cellular changes in tissue, we performed flow cytometry at the same time points as those that were used for RNA isolation. When we examined the flow cytometry data for NKT cells, as expected, we observed overall reduced numbers of NKT cells in the spleen with ketotifen treatment (Fig. 6A and B), but consistent with the expression data, increased numbers of these NKT cells were CD44^+^ (Fig. 6C and D). This suggests that although ketotifen reduced inflammation in the spleen, it did not prevent NKT cells from becoming activated. There was little change in the numbers of NK cells predicted by the DCQ algorithm on day 1, but by day 3, this analysis suggested that there were many fewer NK cells in ketotifen-treated mice (Fig. 5A). Flow cytometry analysis revealed that numbers of NK cells were significantly reduced on both day 1 (data not shown) and day 3 (Fig. 6A) after infection in ketotifen-treated mice compared to vehicle-treated mice. Similarly, there was a reduction in the number of NK cells expressing CD4 (an activation marker for NK cells) after ketotifen treatment (Fig. 6E and F). This result is consistent with our previous observation that MC activation results in the recruitment of NK and NKT cells (39), since here, blocking of MC activation by using ketotifen decreased the number of NK cells in the spleen. DCQ analysis also revealed perturbations in T cell subsets, consistent with the transition to adaptive immune responses that we expected to occur between days 1 and 3 of infection. In comparison to vehicle-treated DENV-infected mice, there were overall reduced total numbers of total CD8^+^ T cells in the spleens of ketotifen-treated mice (Fig. 6G and H). Thus, the results of flow cytometry showed generally consistent relative changes in cell populations compared to the expression data, but DCQ analysis sensitively detected changes in genes associated with certain reference populations. Overall, ketotifen-treated mice have reduced inflammation in the spleen but do not appear to have a reduced capacity for the activation of key innate immune cell types that are responsible for clearing infection, such as NK cells and NKT cells.

## DISCUSSION

Despite the prevalence of DENV disease in tropical and subtropical regions across the globe, there are no approved antivirals or therapeutics for its treatment. Treatment design is complicated by the fact that severe disease tends to occur during the defervescence stage of the illness after the virus has been cleared. As such, it is necessary to understand the host drivers of DENV disease when devising treatment strategies. Our paper presents the first characterization of the host transcriptomic response to DENV infection in an immunocompetent animal model. Our mouse model of DENV infection recapitulates host responses observed in humans. We also show that ketotifen, a DENV therapeutic candidate that is currently in clinical trials (ClinicalTrials registration number NCT02673840 [https://clinicaltrials.gov/show/NCT02673840]), reduces the aberrant host responses that drive DENV disease severity without having a broadly immunosuppressive effect.

Our data indicate that the C57BL/6 mouse host response to infection with the DENV EDEN2 strain recapitulates the human host response to DENV. We found that DENV infection disrupted metabolic pathways and promoted an inflammatory environment in the spleen and liver, key DENV target organs. DENV infection perturbs lipid and carbohydrate metabolism pathways in humans and human cell lines (10, 37). Liver impairment is a common outcome in DENV-infected patients and results in changes in levels of serum metabolites such as very-low-density lipoprotein (VLDL) and low-density lipoprotein (LDL) (10) and liver health markers such as aspartate aminotransferase (AST) and alanine aminotransferase (ALT) (45). We identified DENV-induced upregulation of macromolecular metabolism genes such as NAMPT, SREBF1, and LPIN1 in C57BL/6 mouse livers. Increased levels of SREBF1 and LPIN1 are associated with the increased uptake of lipids in the liver (46–48). These metabolic changes may partially explain the liver dysfunction seen in dengue fever (DF) and DHF/DSS patients (10, 11, 45).

A DENV-induced inflammatory response was indicated by increases in the expression levels of anti-DENV ISGs such as ISG15 and OAS and cytokine genes such as CCL2, CCL4, IL-15, and CXCL10 in C57BL/6 mouse spleens. Anti-DENV ISGs and CXCL10 were also upregulated in the liver. CCL2 and CCL4 act as T cell chemoattractants (49, 50). CCL2 also stimulates MC activation (51). IL-15 induces NK cell and memory CD8^+^ T cell proliferation (52–54), while CXCL10 promotes the formation and trafficking of effector T cells (55). Transcripts and proteins corresponding to these cytokines are also upregulated in humans infected with DENV (11, 12, 14, 36, 37, 41, 56–58). There are functional consequences of the upregulation of these cytokines. DENV infection leads to NK and T cell expansion and activation in humans with dengue fever (42, 43, 59, 60). It also leads to increases in T and NK cell recruitment to the spleen in our model. Previously, we observed that MCs increase the recruitment of NK and NKT cells to skin sites of DENV infection (39). While MC activation and recruitment appear to be a protective response to localized infection in the skin by promoting the clearance of DENV, the role of NK cells in systemic infection is less clear, and some studies have shown that increased numbers of these cells in the blood are associated with more severe disease (24, 25).

MCs, the specific target of ketotifen, are important mediators of DENV disease (24, 25, 61). They release both inflammatory and vasculature-regulating molecules during DENV infection and promote vascular leakage in infected animals. Inhibition of MC degranulation by ketotifen inhibits DENV-mediated vascular leakage (24, 25). Ketotifen reduced the upregulation of inflammatory genes such as CXCL10 in the liver and CXCL10 and CCL2 in the spleen. However, the reduction in cytokine transcription was not uniform. Ketotifen had no effect on IL-15 levels, and ketotifen actually induced CCL17 and IL-18 gene expression in the spleen. CCL17 is a CD4^+^ T cell chemoattractant (62), while IL-18 activates NK and NKT cells (63, 64). Thus, although ketotifen suppressed the function of MCs as expected, it was not broadly immunosuppressive. This is further evidenced by the expression of major histocompatibility complex (MHC) and costimulatory molecules in the spleens of ketotifen-treated animals, supporting that the antigen presentation machinery was not suppressed by treatment.

Immunosuppressive drugs such as corticosteroids have been tested in clinical trials, but contradictory results have made it impossible to tell if they are effective DENV therapeutics (65). The side effects of corticosteroid treatment are also cause for concern. For example, corticosteroids induce T cell apoptosis (66, 67). High-dose acute treatment with corticosteroids can also reduce serum IgG titers (68). As such, therapeutics that specifically target the effects of DENV immunopathology but preserve the beneficial aspects of the immune response are more desirable. MC stabilization could have off-target effects and may also reduce the release of mediators from cells such as macrophages (69), but in general, this strategy is highly targeted and less immunosuppressive and would not have the strong inhibitory effects on T cells that result from steroid use. MC stabilization allows the immune system to clear infection while also inhibiting proinflammatory responses that are associated with vascular leakage, including that during DENV infection (24, 70). Theoretical off-target effects of ketotifen on monocytes and macrophages could also be beneficial since these cells are sources of vasoactive cytokines and also amplify DENV replication *in vivo* as target cell types for infection (71, 72). The ability to sensitively detect the specific host pathways that are perturbed by a drug candidate, as we have done here, can mitigate concerns about using immunomodulatory drugs to treat DENV infection in humans. Furthermore, this approach can identify gene expression signatures that can serve as correlates of drug efficacy in clinical trials.

We have shown that ketotifen has a variety of effects on the host response to DENV. It reduced the response to DENV in the liver and the spleen by dampening the differential expression of approximately half of the DENV-induced genes. In addition to reversing DENV-induced host signatures, ketotifen also influenced the LXR/RXR pathway and reduced the transcription of genes involved in cholesterol biosynthesis in the liver. Interestingly, decreases in serum cholesterol and LDL levels also occur during DF and DHF (10, 73), and DENV infection stimulates cellular cholesterol uptake to stimulate the synthesis of fatty acids that it uses for replication (74–76). MC activation causes macrophages to take up cholesterol by a mechanism that is dependent on the phagocytosis of cellular granules released from degranulated MCs (77). This presents the intriguing possibility that ketotifen treatment negatively impacts DENV replication as well. DENV-mediated vascular leakage decreases exponentially with ketotifen treatment (24, 25). One might expect an exponential increase in DENV levels upon ketotifen treatment, but the increase is small and subsequently cleared by the immune system (24, 25). Although previous studies have shown that MCs promote the overall clearance of the virus (39), further investigation is needed to understand whether there are unique influences of MC products on virus replication in different cell types.

Our study is the first report of full-genome transcriptional analysis using an MC-stabilizing drug *in vivo* against DENV in an experimental system. Our *in silico* predictions and *in vivo* validation emphasize the contributions made by MCs to DENV disease that can be therapeutically reversed by using MC-stabilizing drugs such as ketotifen. This study provides the field with a unique global transcriptional map that results from MC stabilization as well as an understanding of the molecular mechanisms that MCs utilize to influence dengue disease outcomes. Our results indicate the suitability of MC-targeting drugs as candidates for the treatment of DENV and other syndromic diseases.

## MATERIALS AND METHODS

### Animal studies.

C57B/6NTac mice were purchased from InVivos, Singapore, and housed in the Duke-National University of Singapore (Duke-NUS) vivarium. Mice were infected with 1 × 10^6^ PFU of DENV2 strain EDEN2 by i.p. injection in 100 μl of phosphate-buffered saline (PBS). Drug-treated animals were given 0.6 mg per mouse per day of ketotifen (Sigma) or an equivalent volume of saline by i.p. injection, as previously optimized (24). The first dose was administered 1 h after infection, with daily drug injections thereafter at 24-h intervals. DENV was propagated in c6/36 cells and titrated by using standard methods, as previously described (39).

### RNA isolation and microarray processing.

RNA extraction from tissues of virus- and mock-infected C57B/6NTac mice was performed in triplicate. Probe labeling and microarray slide hybridization for each biological replicate were performed by using the Mouse Whole Genome Microarray 4x44K kit (Agilent Technologies) according to the manufacturer's instructions. Slides were scanned on an Agilent DNA microarray scanner (model G2505B) using the XDR setting, and raw images were analyzed by using Agilent Feature Extraction software (version 9.5.3.1). Extracted raw data were partitioned into liver and spleen samples and separately background corrected by using the “norm-exp” method with an offset of 1 and quantile normalized by using the limma package in the R environment (78). Probes were filtered for low intensity, requiring at least two samples with intensity above a threshold set at the 5% quantile for intensity. This resulted in 32,986 probes retained for the liver samples and 29,608 probes retained for the spleen samples. Probes were mean-summarized by gene.

### Viral replication.

RNA was isolated by using the TRIzol/RNeasy hybrid RNA isolation protocol. For the detection of the negative strand, cDNA was synthesized by using an iScript Select cDNA synthesis kit using a sense primer, C14A (AATATGCTGAAACGCGAGAGAAACCGCG), followed by PCR with primer pair C14A and C69B (5′-CCCATCTCITCAIIATCCCTGCTGTTGG-3′), to amplify a 170-bp region from the capsid-PrM region of the DENV genome (79, 80). For the quantification of total DENV genome copy numbers, cDNA was synthesized by using primer C69B and an iScript cDNA synthesis kit, and quantitative real-time PCR was performed by using primer pair C14A and C69B and probe VICD2C38B (AGC ATT CCA AGT GAG AAT CTC TTT GTC AGC TGT) (80).

### Identification of differentially expressed genes.

Differential expression was determined by comparing DENV-infected replicates to mock-infected samples based on a linear model for each gene by using limma. Criteria for differential expression were an absolute log_2_ fold change (FC) of 0.58 and an adjusted *P* value of 0.05, calculated by using a moderated *t* test with subsequent Benjamini-Hochberg correction. Between the DENV2- and mock-infected liver samples, 192 upregulated DE genes and 128 downregulated DE genes were identified at day 1, and 8 upregulated DE genes and 1 downregulated DE gene were identified at day 3. Between the DENV2-infected, ketotifen-treated and mock-infected liver samples, 846 upregulated DE genes and 804 downregulated DE genes were identified at day 1, and 7 upregulated DE genes and 1 downregulated DE gene were identified at day 3. Between the DENV2-infected and mock-infected spleen samples, 213 upregulated DE genes and 7 downregulated DE genes were identified at day 1, and 55 upregulated DE genes and 6 downregulated DE genes were identified at day 3. Between the DENV2-infected, ketotifen-treated and mock-infected spleen samples, 667 upregulated DE genes and 374 downregulated DE genes were identified at day 1, and 52 upregulated DE genes and 11 downregulated DE genes were identified at day 3.

### Functional enrichment.

Functional analysis of statistically significant gene expression changes was performed by using the Ingenuity Pathway Analysis Knowledge Base (IPA; Ingenuity Systems). For all gene set enrichment analyses, right-tailed Fisher's exact test was used to calculate the probability that the enrichment of each biological function was due to chance alone. All enrichment scores were calculated by IPA, using the probes that passed our quality control (QC) filter as the background data set.

### Computational measurement of immune cell subsets.

To infer the immune cell quantities for each DENV2- and mock-infected sample, we used the decomposition-based digital cell quantification (DCQ) algorithm (81). The algorithm utilizes an immune cell compendium of transcriptional profiles for 207 isolated immune cell subsets and the signatures of 61 predefined gene surface markers discriminating these cell types. Given the relative expression across each gene from the microarray samples, DCQ models differential expression as the sum of changes in quantities of the 207 immune cell types by using an “elastic net” regression technique and the signatures of the gene markers. The DCQ output reflects relative immune cell quantities for each immune cell subtype. Ten output models were generated, and relative cell quantities were taken from their average. A lambda minimum, a parameter of L1 and L2 regularization, of 0.2 was used.

### Quantification of splenic cells by flow cytometry.

Spleens were harvested, dissociated with collagenase (Sigma), and processed to single-cell suspensions by using a 70-μm cell strainer (BD Biosciences). Total cell numbers were quantified by using a hemocytometer. Cells were then stained with the following antibodies: anti-CD45-BUV395, anti-CD3e-peridinin chlorophyll protein (PerCP)-Cy5.5, anti-CD4-BV650, anti-CD8a-Alexa 700, anti-CD62L-phycoerythrin (PE)-Cy7, anti-CD44-BV510 (all from BD Biosciences), and anti-NK1.1-PE (BioLegend). Flow cytometry data were acquired with an LSRFortessa cell analyzer (BD Biosciences) and analyzed by using FlowJo software (FlowJo, LLC).

### Ethics statement.

The SingHealth Institutional Animal Care and Use Committee approved animal protocols (2012/SHS/774) in accordance with the National Advisory Committee for Laboratory Animal Research (NACLAR).

### Data availability.

All primary expression microarray data have been deposited in the NCBI Gene Expression Omnibus (accession number GSE100196).

## Supplementary Material

Supplemental material
