# Supplementary material for: Transcriptional Profiling Confirms the Therapeutic Effects of Mast Cell Stabilization in a Dengue Disease Model
Source: J Virol. 2017 Aug 24;91(18):e00617-17. doi: 10.1128/JVI.00617-17 (PMC5571258; doi:10.1128/JVI.00617-17)
Supplement: Supplemental material [file supp_91_18_e00617-17__index.html]

Supplemental material 

# Transcriptional Profiling Confirms the Therapeutic Effects of Mast Cell Stabilization in a Dengue Disease Model

## Supplemental material

- Supplemental file 1 -

  Fig. S1 (Validation of DENV infection of spleen and liver tissues.)

  Fig. S2 (Ketotifen treatment partially reverses the DENV2-mediated host response in the liver.)

  Fig. S3 (Ketotifen treatment partially reverses the DENV2-mediated host response in the spleen.)

  PDF, 881K
