# Supplementary material for: Transcriptional Profiling Confirms the Therapeutic Effects of Mast Cell Stabilization in a Dengue Disease Model
Source: J Virol. 2017 Aug 24;91(18):e00617-17. doi: 10.1128/JVI.00617-17 (PMC5571258; doi:10.1128/JVI.00617-17)
Supplement: Supplemental material [file JVI.00617-17_zjv999182884s1.pdf]

Supplemental Material

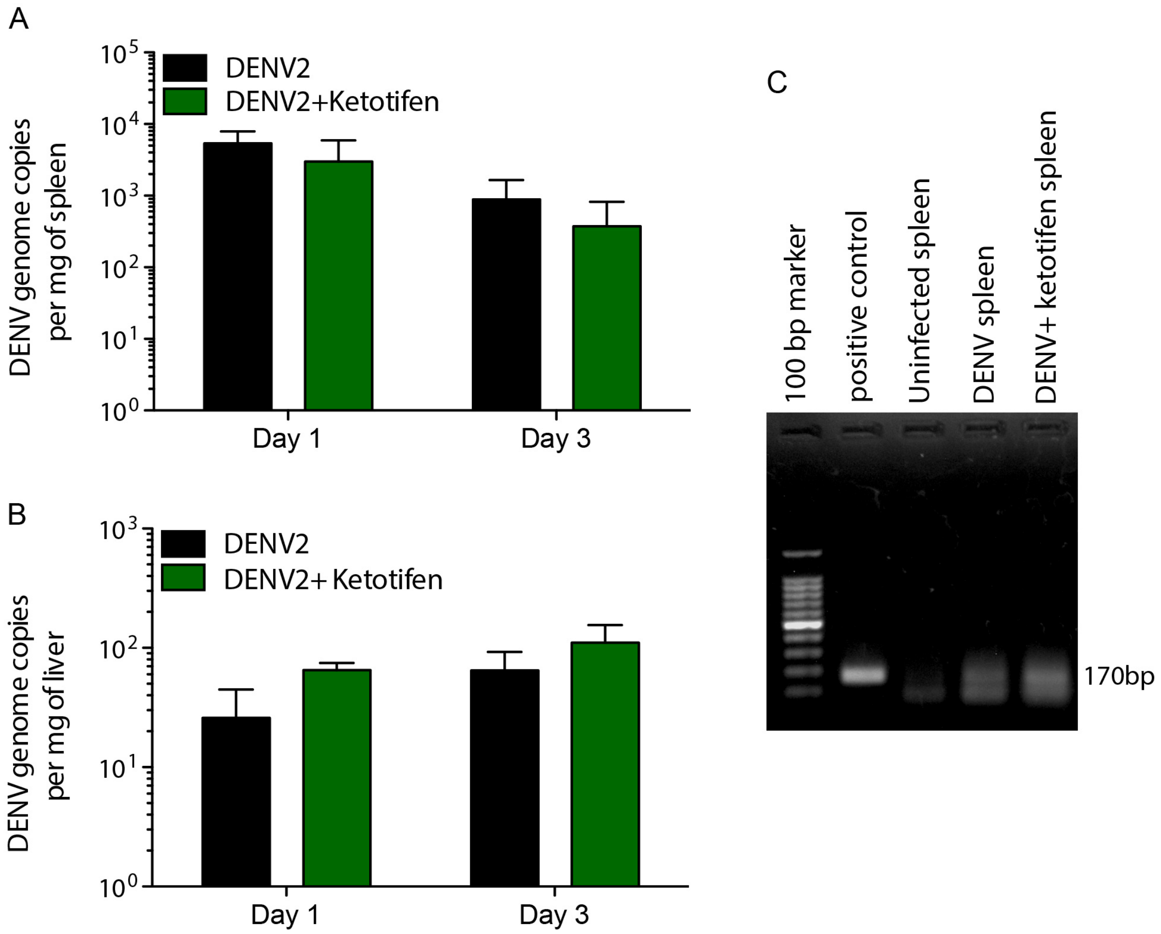

Supplemental Figure 1 - Validation of DENV infection of spleen and liver tissues. Virus genome copies were quantitated in the (**A**) spleen and (**B**) liver of DENV-infected and ketotifen-treated mice days 1 and 3 post-infection. Viral copy numbers did not differ significantly by 2-way ANOVA. (**C**) Uncut gel image demonstrating that active virus replication was confirmed by measuring DENV negative-strand RNA using spleen samples from day 1 post-infection.

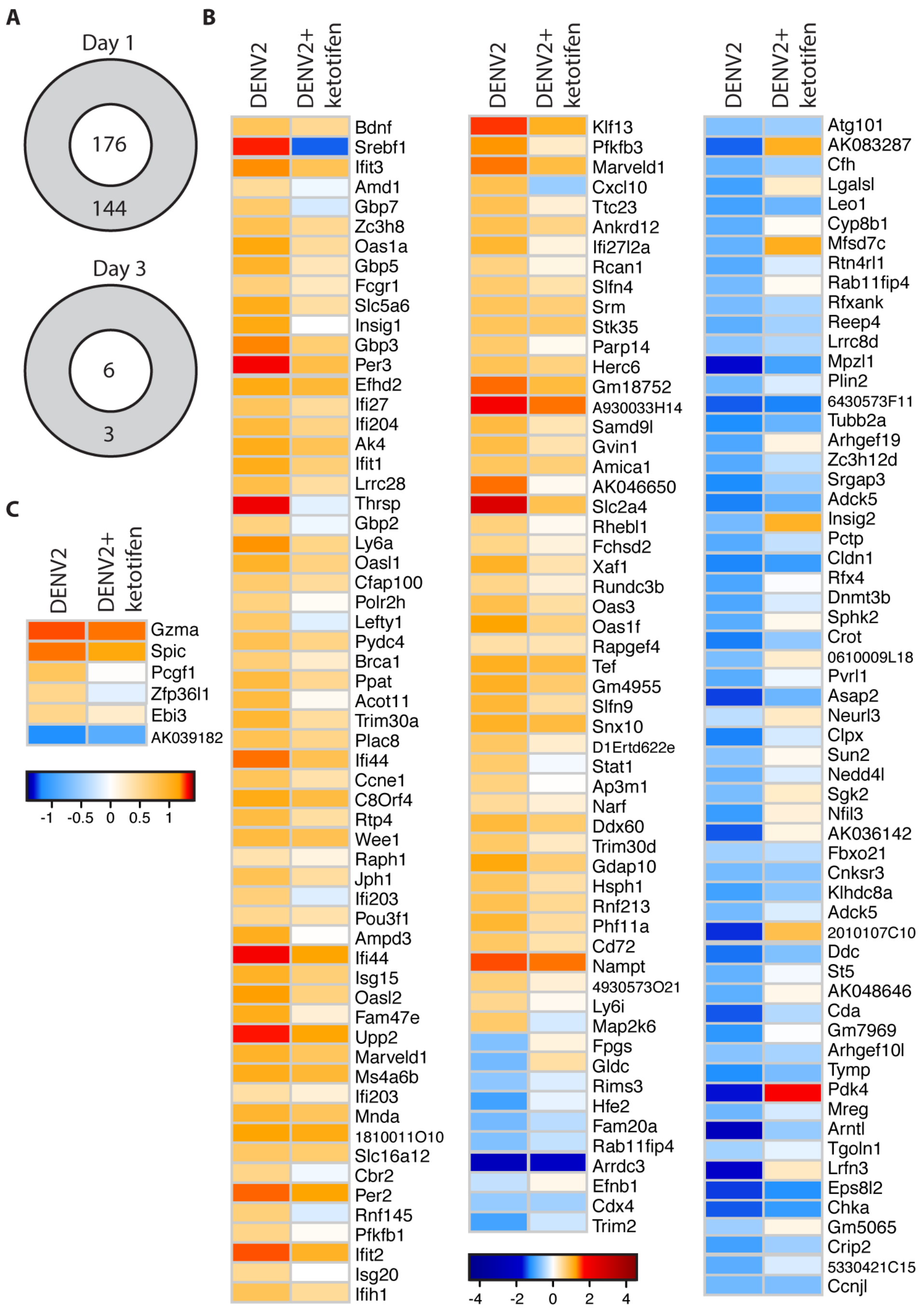

Supplemental Figure 2 - Ketotifen treatment partially reverses the DENV2-mediated host response in the liver. (**A**) Venn diagrams showing the number of DENV2-induced DE genes in the liver on days 1 and 3 whose expression changes are reversed by at least 10% by ketotifen treatment. Heatmaps showing the DENV2-induced genes in the liver whose expression changes on (**B**) day 1 and (**C**) day 3 are reversed by at least 10% by ketotifen treatment.

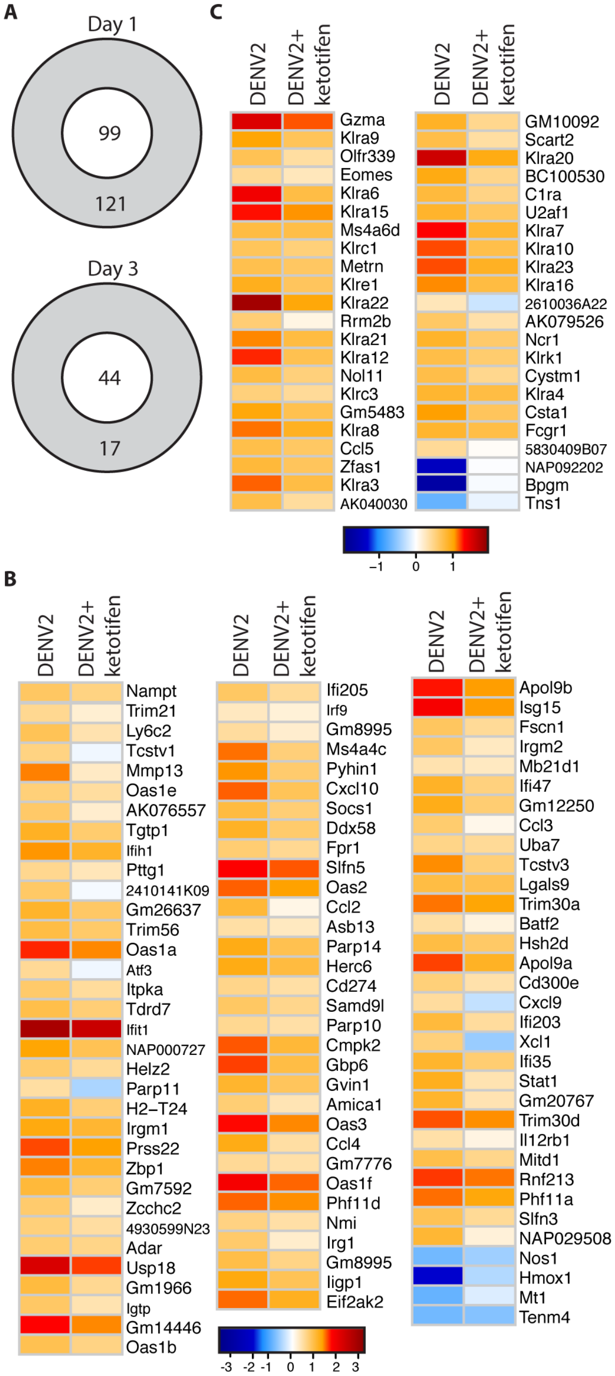

Supplemental Figure 3 - Ketotifen treatment partially reverses the DENV2-mediated host response in the spleen. (**A**) Venn diagrams showing the number of DENV2-induced DE genes in the spleen on days 1 and 3 whose expression changes are reversed by at least 10% by ketotifen treatment. Heatmaps showing the DENV2-induced genes in the spleen whose expression changes on (**B**) day 1 and (**C**) day 3 are reversed by at least 10% by ketotifen treatment.
